# Age-Related Disparity in Immediate Prognosis of Patients with Triple-Negative Breast Cancer: A Population-Based Study from SEER Cancer Registries

**DOI:** 10.1371/journal.pone.0128345

**Published:** 2015-05-28

**Authors:** Wenjie Zhu, Edith A. Perez, Ruoxi Hong, Qing Li, Binghe Xu

**Affiliations:** 1 Department of Medical Oncology, Cancer Hospital, Chinese Academy of Medical Sciences and Peking Union Medical College, Beijing, China; 2 Mayo Clinic, Jacksonville, Florida, United States of America; Taipei Medical University, TAIWAN

## Abstract

**Background:**

Triple-negative breast cancer (TNBC) has been demonstrated to carry poor prognosis, but whether or not there exists any age-related variation in TNBC outcomes has yet to be elucidated. The current population-based study investigated the early survival pattern of elderly women with TNBC and identified outcome-correlated factors.

**Patients and Methods:**

We searched the Surveillance, Epidemiology, and End Results (SEER) database and enrolled female primary non-metastatic TNBC cases. The patients were subdivided into elderly (≥70 years) and young groups (<70 years). The survival status of elderly patients was compared to that of the younger women. The primary and secondary endpoints were cancer-specific survival (CSS) and overall survival (OS) respectively.

**Results:**

9908 female TNBC patients diagnosed from 2010 to 2011 were included in the current study (20.4% elderly). Elderly patients with relatively advanced diseases exhibited distinctly worse cancer-specific (log-rank, p<0.001) and overall survival (log-rank, p<0.001) than their young counterparts. Advanced age at diagnosis (≥70 years) was significantly predictive of poor outcome in terms of CSS (hazard ratio (HR), 2.125; 95% confidence interval (CI), 1.664 to 2.713; p<0.001) and OS (HR, 3.042; 95%CI, 2.474 to 3.740; p<0.001). Underuse of curative treatment especially radiotherapy was more prevalent in elderly women with stage II or III diseases than in younger patients.

**Conclusion:**

Elderly patients with TNBC displayed elevated early mortality within the first two years of diagnosis compared to the younger individuals. The observed lower rate of loco-regional treatment might be associated with worse cancer-specific outcome for these patients.

## Introduction

As the most common incident form of female cancer worldwide, breast cancer has constituted a considerable disease burden to public health [1]. Triple-negative breast cancer (TNBC), which can be referred to as the tumors that display absence of estrogen receptor (ER) and progesterone receptor (PR) without the overexpression of human epidermal growth factor receptor 2 (HER2), accounts for 15–20% of breast cancer [2, 3]. TNBC is associated with younger age at diagnosis, African-American race, higher grade and more advanced disease stage [4–7]. TNBC is characterized by more aggressive metastases which tend to involve viscera such as lungs and brain rather than bones [8]. A 60–70% overlap exists between TNBC and basal-like breast cancer, which represents one of the five intrinsic molecular subtypes, but they are not completely identical [3, 9].

It has been well established that TNBC carries poor prognosis with an early peak of recurrence at 2–3 years after initial surgical excision and compromised 5-year survival rate when compared to other subtypes [10, 11], but whether or not there exists any age-related variation in TNBC outcomes has yet to be elucidated. Few studies are available exploring the different prognoses of younger and older TNBC patients. Limited evidence has provided controversial data related to the impact of age on TNBC management and outcome. In a retrospective study of 653 TNBC patients Thike et al found that younger age which was defined as below the mean age of the study population (53 years old) was associated with worse disease-free survival (DFS) and overall survival (OS) [12]. However, Cheung et al observed no significant difference in 5-year local recurrence and survival rate between old-aged (≥70 years old) and young TNBC patients under the circumstance that the old-aged group didn’t receive any adjuvant chemotherapy [13]. It is estimated that approximately 21% of breast cancer cases are diagnosed at an advanced age of greater than 70 years [14]. Although breast cancer in elderly individuals is characterized by more favorable tumor biology with a high proportion of hormone receptor (HR)-positive and HER2-negative tumors [15], 15% of older women are still exposed to the risk of developing TNBC [16].

Given the well-known fact that the majority of TNBC recurrence cases occur within the first 2–3 years of diagnosis and inadequate attention has been paid to the immediate survival of TNBC patients following disease relapse, it’s justified to focus on the short-term survival status after primary diagnosis. In the current population-based research, we sought to investigate the early survival pattern of elderly women with TNBC within two years of initial diagnosis and identify any outcome-correlated factors.

## Patients and Methods

### Database

The Surveillance, Epidemiology, and End Results (SEER) Program of the National Cancer Institute (NCI) collects cancer incidence and survival data from US population-based cancer registries. The SEER 18 registries which consist of the SEER 17 (Atlanta, Connecticut, Detroit, Hawaii, Iowa, New Mexico, San Francisco–Oakland, Seattle‐Puget Sound, Utah, Los Angeles, San Jose‐Monterey, Rural Georgia, the Alaska Native Tumor Registry, Greater California, Kentucky, Louisiana, and New Jersey) plus Greater Georgia and cover cases diagnosed from 1973 to 2011 were included in our research [17].

### Study Population

We enrolled female primary breast cancer patients who were classified as having TNBC at diagnosis based on local reporting. Stage IV cases and those who were complicated by multiple malignancies were excluded from the present analysis. Inflammatory breast cancer and cases that recorded unknown ER, PR and/or HER2 status were further rejected. Statistics about HER2 status were not available in SEER database until 2010, and the most recent release of SEER research data on April 15, 2014 has been updated to cover cases diagnosed from 1973 to 2011. Therefore the study population only encompassed TNBC patients who were diagnosed with malignancy from January 2010 through December 2011 and whose tumors had been evaluated for the 3 relevant markers.

Survival data and relevant case-specific information such as age at diagnosis, histological grade, tumor size, lymph node metastasis, AJCC stage, surgery and radiotherapy were retrospectively gathered and compiled. Patients were subdivided into the young (<70 years) and old-aged (≥70 years) groups according to age at diagnosis. Follow-up data were available monthly until December 31, 2011. SEER database released research data about survival status and cause of death but failed to specify any information about local/distant relapse. As a consequence, the primary endpoint was cancer-specific survival (CSS), which was defined as the duration from initial diagnosis to breast cancer-related death. OS was set as the secondary outcome.

Since the present study is a database-dependent analysis rather than experimental research on humans, approval of ethics committees and consent from participating patients are not needed. Patient records/information was anonymized and de-identified prior to analysis. Our access to SEER cancer registries was granted by SEER program after we signed Data-Use Agreement for the SEER 1973–2011 Research Data File.

### Statistical Analysis

The clinical and pathological characteristics of incorporated cases were compared between the two age groups using Chi square test. In survival analysis Kaplan-Meier method was adopted to depict the survival curves, with log-rank test being performed to detect any significant difference in survival distribution. Univariate and multivariate analyses by Cox proportional hazards regression and logistic regression were carried out in order to determine the outcome-related elements. Two-sided p-values were reported and p<0.05 was considered statistically significant. All analyses were done utilizing SPSS version 20.0 software package (SPSS Inc., Chicago, Ill, USA).

## Results

### Baseline Characteristics of Study Population

In all, 9908 female TNBC patients were enrolled in the current study, with 2017 cases older than 70 years of age accounting for 20.4% of total records, which corroborated previous finding about the proportion of elderly patients in overall breast cancer burden (21%) [14]. The median age was 77 and 53 years for older-aged (70–100 years) and younger individuals (15–69 years) respectively. Table 1 showed the major baseline characteristics of the research cohort. Notable age-related difference was detected in all relevant clinical and pathological variables. Compared with the younger patients, older-aged TNBC women tended to develop tumors that harbor a benign biological phenotype, such as lower probability of lymph node metastasis (N1-3, 30.5% *v* 36.2%; p<0.001), earlier TNM stage (stage I, 42.5% *v* 35.2%; p<0.001) and better differentiation (grade I/II, 28.4% *v* 17.0%; p<0.001). Therapy-concerning indexes such as the proportion of surgery and radiation generated cues as to the present situation of clinical management of elderly TNBC cases. 94.6% of patients less than 70 years old underwent some form of surgical excision while 92.8% of older women underwent surgery (p = 0.002). As for radiation a similar trend was observed, with less than a half of old patients being given radiotherapy (41.6% *v* 50.8%; p<0.001).

**Table 1:** Baseline characteristics of study population.

N /%		Young group (<70 years)		Old-aged group (≥70 years)		All		p value ^a^
**Tumor size**	**T0**	23	0.3%	5	0.3%	28	0.3%	**<0.001**
	**T1**	3290	42.7%	948	48.6%	4238	43.9%	
	**T2**	3352	43.5%	712	36.5%	4064	42.1%	
	**T3**	693	9.0%	141	7.2%	834	8.6%	
	**T4**	350	4.5%	145	7.4%	495	5.1%	
**Lymph node metastasis**	**N0**	4980	63.8%	1367	69.5%	6347	64.9%	**<0.001**
	**N1**	2003	25.7%	376	19.1%	2379	24.3%	
	**N2**	491	6.3%	125	6.4%	616	6.3%	
	**N3**	334	4.3%	98	5.0%	432	4.4%	
**TNM stage**	**I**	2708	35.2%	824	42.5%	3532	36.6%	**<0.001**
	**II**	3677	47.8%	756	39.0%	4433	46.0%	
	**III**	1315	17.1%	358	18.5%	1673	17.4%	
**Grade**	**I**	150	2.0%	62	3.2%	212	2.2%	**<0.001**
	**II**	1134	15.0%	487	25.2%	1621	17.1%	
	**III**	6208	82.0%	1367	70.9%	7575	79.7%	
	**IV**	80	1.1%	13	0.7%	93	1.0%	
**Surgery**	**No**	424	5.4%	145	7.2%	569	5.8%	**0.002**
	**Yes**	7457	94.6%	1867	92.8%	9324	94.2%	
**Radiation**	**No**	3677	49.2%	1142	58.4%	4819	51.1%	**<0.001**
	**Yes**	3803	50.8%	815	41.6%	4618	48.9%	
**Survival status (cancer-specific)**	**Alive**	7674	97.3%	1897	94.1%	9571	96.6%	**<0.001**
	**Dead**	217	2.7%	120	5.9%	337	3.4%	
**Survival status (overall)**	**Alive**	7628	96.7%	1816	90.0%	9444	95.3%	**<0.001**
	**Dead**	263	3.3%	201	10.0%	464	4.7%	

^a^ Two-sided p values by chi-squared test with p<0.05 being statistically significant.

### Survival Analysis

As showcased in Table 1, in the older-aged group (n = 2017), 201 deaths were reported among which 120 deaths were attributable to breast cancer, leading to a total mortality rate of 10.0%, which is markedly higher than that of the younger patients (3.3%; p<0.001). When it came to cancer-specific mortality similar result was obtained, with more elderly patients dying of TNBC (5.9% *v* 2.7%; p<0.001).

**Fig 1:**
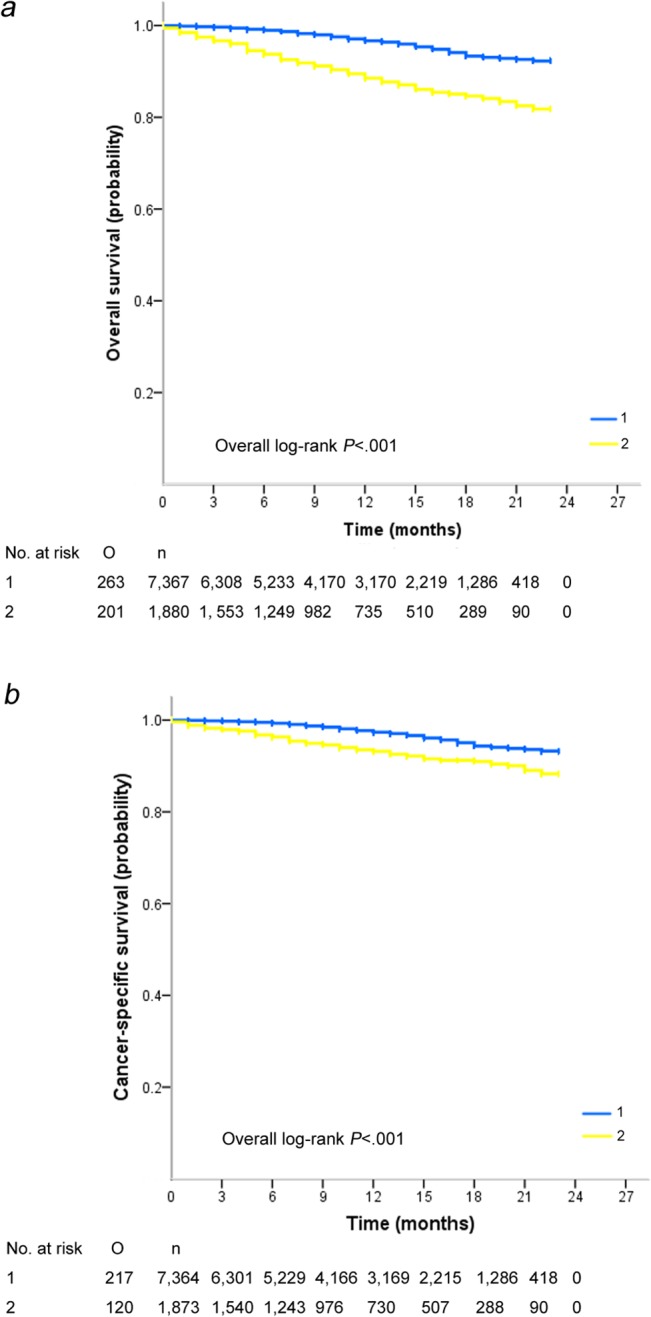
Overall (A) and cancer-specific survival (B) of young (group 1) and elderly (group 2) patients with TNBC. n, number of patients; O, observed events.

Fig 1 presented the survival distribution of two age groups. Elderly TNBC cases were associated with distinctly worse overall (log-rank p<0.001; Fig 1A) and cancer-specific survival (log-rank p<0.001; Fig 1B) than their young counterparts. The 18-month overall survival rate was 84.6% in the elderly group compared with 93.4% in the younger group. The 18-month cancer-specific survival rates were 91.0% and 94.3% in the elderly and younger groups respectively, which suggested a survival disadvantage related to older age. Cox proportional hazards regression method was then applied to explore the prognosis-concerning variables. In Table 2, we describe that after correcting for confounding factors, advanced age at diagnosis (≥70 years) was independently predictive of poor outcome in terms of CSS and OS (for CSS, HR, 2.125; 95%CI, 1.664 to 2.713; p<0.001; for OS, HR, 3.042; 95%CI, 2.474 to 3.740; p<0.001). Tumor-related elements such as tumor size, lymph node metastasis and AJCC stage were found to be effective in distinguishing outcomes as well. Of importance, loco-regional treatment modalities including surgery (for CSS, HR, 0.250; 95%CI, 0.186 to 0.337; p<0.001; for OS, HR, 0.291; 95%CI, 0.220 to 0.384; p<0.001) and radiation (for CSS, HR, 0.504; 95%CI, 0.390 to 0.651; p<0.001; for OS, HR, 0.439; 95%CI, 0.348 to 0.552; p<0.001) both exerted profound impact on TNBC prognosis.

**Table 2:** Outcome-related factors in triple-negative breast cancer by multivariate analysis.

	Overall survival		Cancer-specific survival	
	HR	p value	HR	p value
**Age at diagnosis**		**<0.001**		**<0.001**
**<70 years**	—		—	
**≥70 years**	3.042		2.125	
**Surgery**		**<0.001**		**<0.001**
**No**	—		—	
**Yes**	0.291		0.250	
**Radiation**		**<0.001**		**<0.001**
**No**	—		—	
**Yes**	0.439		0.504	
**Grade**		**0.040**		0.107
**I-II**	—		—	
**III-IV**	1.376		1.355	
**TNM stages**		**0.001**		**<0.001**
**I**	—		—	
**II-III**	1.843		2.344	
**Tumor size**		**<0.001**		**<0.001**
**T0-2**	—		—	
**T3-4**	3.565		4.327	
**Node metastasis**		**<0.001**		**<0.001**
**N0**	—		—	
**≥N1**	1.759		1.945	

Cox proportional hazards regression model was used. Two-sided p-values were reported with p<0.05 considered statistically significant. Abbreviations: HR, hazard ratio.

### Subgroup Analysis

**Fig 2:**
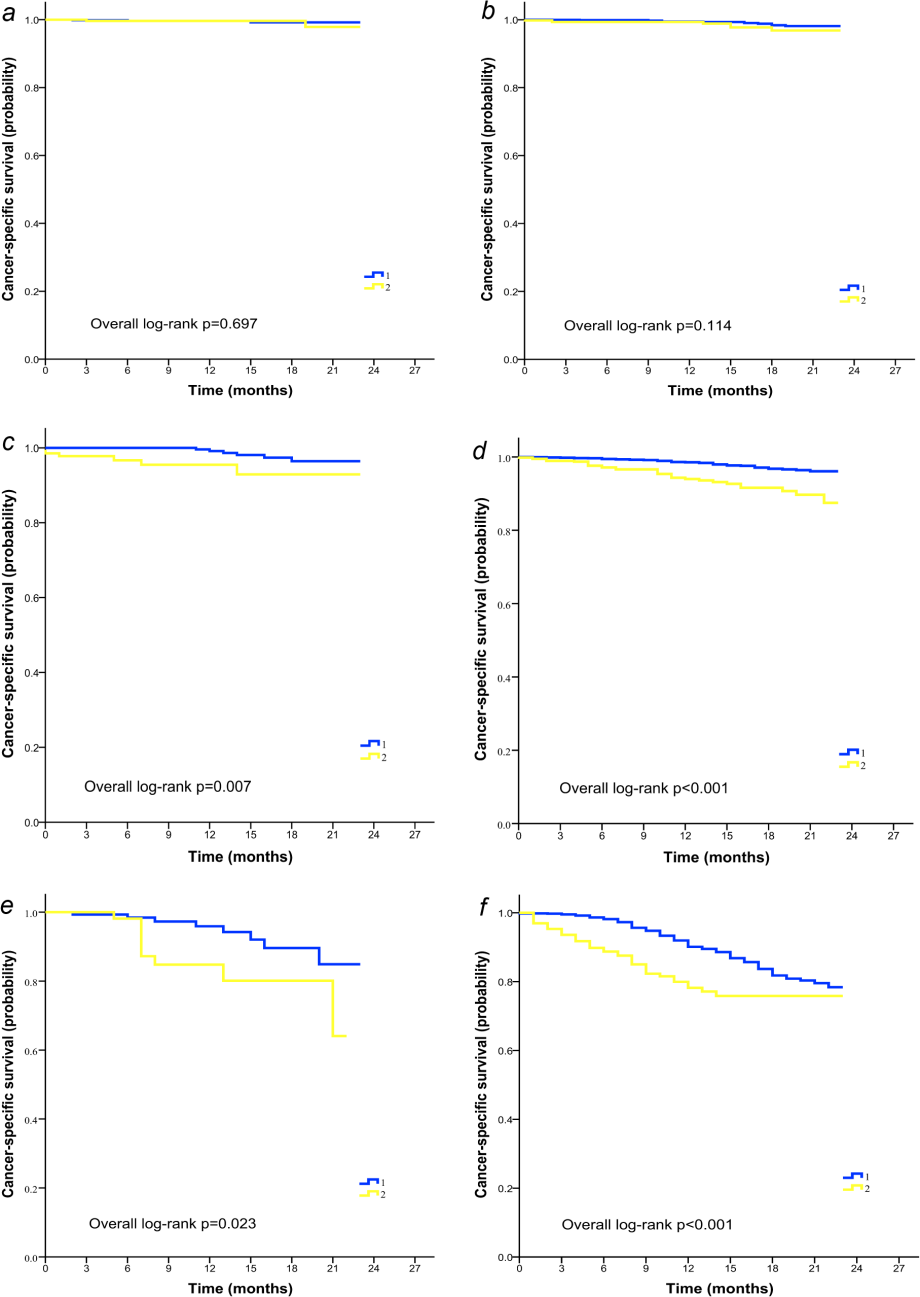
Cancer-specific survival of young (group 1) and elderly (group 2) patients with TNBC stratified by TNM stages and tumor grade. (A) Patients with stage I, grade I-II disease (n = 983). (B) Patients with stage I, grade III-IV disease (n = 2445). (C) Patients with stage II, grade I-II disease (n = 589). (D) Patients with stage II, grade III-IV disease (n = 3681). (E) Patients with stage III, grade I-II disease (n = 214). (F) Patients with stage III, grade III-IV disease (n = 1372).

**Fig 3:**
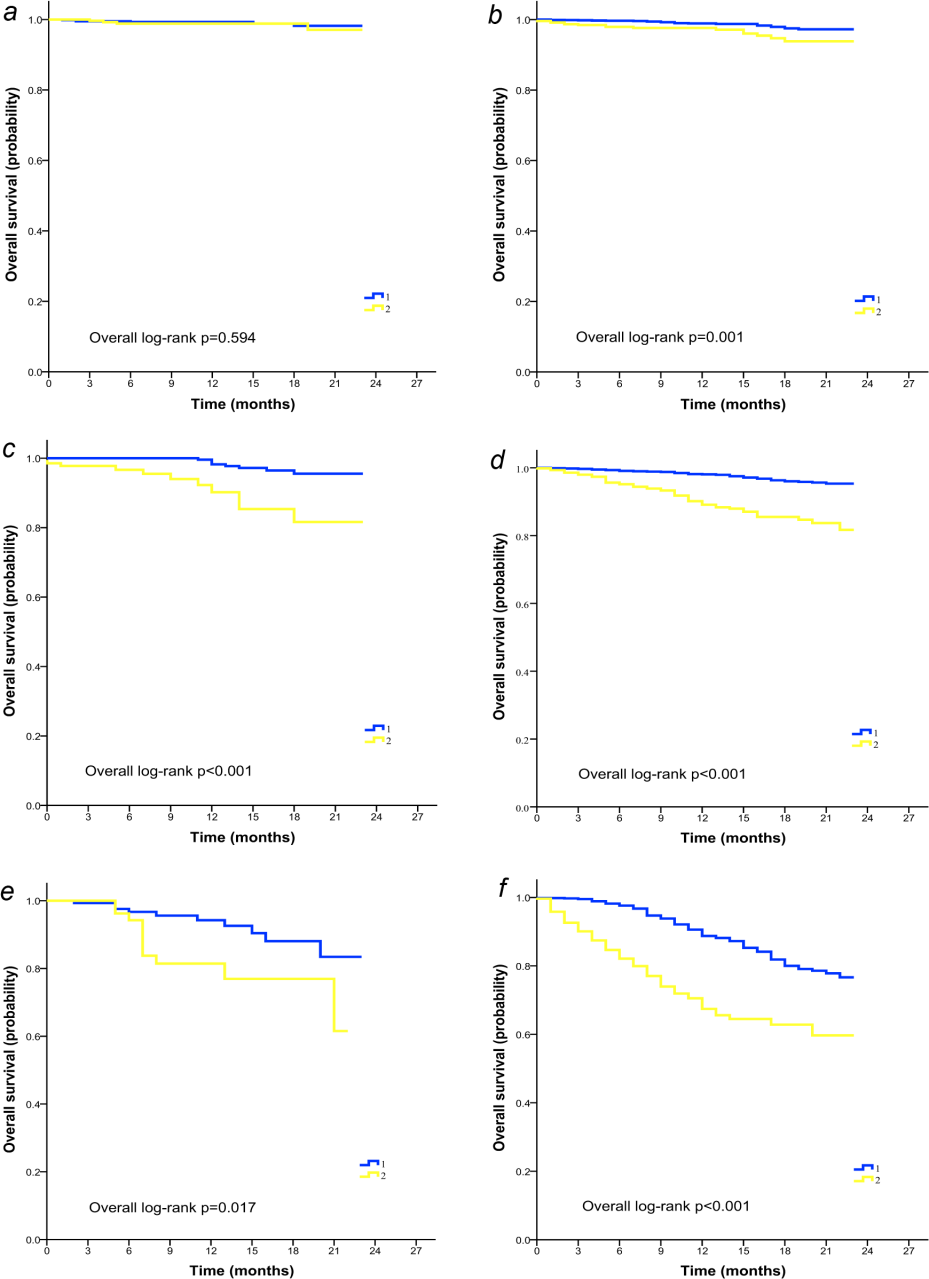
Overall survival of young (group 1) and elderly (group 2) patients with TNBC stratified by TNM stages and tumor grade. (A) Patients with stage I, grade I-II disease (n = 983). (B) Patients with stage I, grade III-IV disease (n = 2445). (C) Patients with stage II, grade I-II disease (n = 589). (D) Patients with stage II, grade III-IV disease (n = 3681). (E) Patients with stage III, grade I-II disease (n = 214). (F) Patients with stage III, grade III-IV disease (n = 1372).

To better understand the impact of age on outcomes of patients with TNBC, further survival analysis was performed stratified by stage of disease and histological grade. It was demonstrated that in subgroups with stage II or III diseases, elderly women displayed inferior CSS (Fig 2C–2F) and OS (Fig 3C–3F) compared with younger patients irrespective of tumor grade. In patients with poorly differentiated (grade III-IV) stage I cancer however, age exerted a differential effect on OS (log-rank p = 0.001; Fig 3B) rather than CSS (log-rank p = 0.114; Fig 2B). Age-associated disparity in survival was not observed in the most benign subtypes of breast cancer (stage I, grade I-II, Fig 2A and Fig 3A).

The records of the older-aged patients (n = 2017) were reviewed for survival analysis. As shown in S1 File, elderly TNBC patients who underwent surgery (n = 1867) had better immediate cancer-specific survival than those who didn’t (n = 145; p<0.001). The aforementioned observation applied to radiation alike. Multivariate analysis demonstrated that surgery (HR, 0.250; 95%CI, 0.153 to 0.409; p<0.001) and radiation (HR, 0.402; 95%CI, 0.244 to 0.662; p<0.001) were both robust prognostic factors in elderly TNBC in addition to tumor size, lymph node metastasis and stage (S1 Table).

Since preliminary exploration disclosed therapy as a prognosis-indicative element, we accomplished further analysis based on the loco-regional treatment status. In the subset of patients who didn’t receive surgical intervention or radiotherapy (n = 460), a statistically different CSS was found between the older and younger TNBC patients (p<0.001; Fig. A in S2 File), with the older-aged group exhibiting inferior survival. As for the patients receiving surgery irrespective of radiation history (n = 9324), age-related divergence in survival remained evident (p<0.001;-Fig. B in S2 File). Similar variation in CSS was also demonstrated in the subgroup that only underwent surgery without radiotherapy (n = 4354; p<0.001; Fig. C in S2 File). However, the constant age-caused gap in survival pattern was considerably narrowed in the TNBC cohort that received comprehensive treatment including both surgery and radiotherapy (n = 4552; p = 0.126; Fig. D in S2 File).

### Patterns of Care in Study Population

The aforementioned analysis revealed the influence of loco-regional treatment on outcomes of patients with TNBC. Hence we further explored the difference in patterns of care between young and elderly patients. As described by Table 3, for patients with stage I or II diseases the rate of surgery in the old-aged group was comparative to that of the young group. A higher proportion of elderly women with stage III TNBC didn’t receive surgical excision compared with the younger patients (14.2% vs 9.1%, p = 0.004 by chi-squared test). As for radiotherapy, for patients with relatively advanced diseases (stage II or III) more than a half of elderly women forewent radiation, resulting in a far lower rate of radiotherapy than that in younger patients (p<0.001 by chi-squared test). In stage I group however, the rates of radiotherapy were quite similar between young and old-aged patients.

**Table 3:** Patterns of care in young (<70 years) and old-aged (≥70 years) patients with triple-negative breast cancer by TNM stage.

N /%	Stage I			Stage II			Stage III		
	Young	Old-aged	p	Young	Old-aged	p	Young	Old-aged	p
**Surgery**			0.825			0.485			**0.004**
**No**	43/1.6	14/1.7		188/5.1	34/4.5		119/9.1	51/14.2	
**Yes**	2664/98.4	810/98.3		3485/94.9	720/95.5		1193/90.9	307/85.8	
**Total**	2707/100	824/100		3673/100	754/100		1312/100	358/100	
**Radiation**			0.931			**<0.001**			**<0.001**
**No**	1221/47.1	377/46.9		1849/53.1	508/69.5		484/39.5	193/55.5	
**Yes**	1370//52.9	426/53.1		1635/46.9	223/30.5		742/60.5	155/44.5	
**Total**	2591/100	803/100		3484/100	731/100		1226/100	348/100	

We subsequently assessed the situation of radiotherapy in patients for whom postoperative radiation should be indicated so as to determine the prevalence of undertreatment in young and elderly groups. Evaluated cases included patients receiving mastectomy with tumor size≥5cm or ≥4 positive lymph nodes and those receiving breast-conserving surgery (BCS). Table 4 displayed that nearly one third of elderly patients failed to undergo radiotherapy following BCS (31.2% vs 26.9%, p = 0.002 by chi-squared test), and a still higher percentage of elderly women at great risk of recurrence didn’t receive radiation after mastectomy when they should have (56.3% vs 36.9%, p<0.001 by Fisher’s exact test). Of note, the registries showed that 2.2% of old-aged patients (n = 21) refused radiotherapy after BCS though the treatment was recommended by doctors. Further analysis stratified by TNM stage (Table 5) disclosed that underuse of radiotherapy was more prevalent in elderly patients with stage II or III diseases than in their younger counterparts (p<0.001 by chi-squared test).

**Table 4:** Use of radiotherapy in patients for whom radiation following breast-conserving surgery or mastectomy should be indicated.

N /%	Breast-conserving surgery			Mastectomy ^a^		
	Young (<70 years)	Old-aged (≥70 years)	p	Young (<70 years)	Old-aged (≥70 years)	p ^b^
**Radiation**			**0.002**			**<0.001**
**No**	977/26.9	295/31.2		379/36.9	156/56.3	
**Refuse**	45/1.2	21/2.2		6/0.6	3/1.1	
**Yes**	2611/71.9	631/66.6		643/62.5	118/42.6	
**Total**	3633/100	947/100		1028/100	277/100	

^a^ Patients for whom post-mastectomy radiotherapy should be indicated include those with≥4 positive lymph nodes or tumor size≥5cm.

^b^ Two-sided p value by Fisher’s exact test.

**Table 5:** Use of radiotherapy in patients for whom radiation following breast-conserving surgery or mastectomy^a^ should be indicated stratified by TNM stage.

N /%	Stage I			Stage II			Stage III		
	Young	Old-aged	p	Young	Old-aged	p	Young	Old-aged	p
**Radiation**			0.535			**<0.001**			**<0.001**
**No**	435/25.0	137/24.8		577/31.9	181/48.7		395/35.5	157/52.5	
**Yes**	1303//75.0	416/75.2		1232/68.1	191/51.3		719/64.5	142/47.5	
**Total**	1738/100	553/100		1809/100	372/100		1114/100	299/100	

^a^ Patients for whom post-mastectomy radiotherapy should be indicated include those with≥4 positive lymph nodes or tumor size≥5cm.

Logistic regression method was adopted in order to disclose any potential factors that might influence the decision of surgery and radiotherapy. In comparison to their younger peers, old-aged TNBC patients were less likely to receive radiation (odds ratio (OR), 0.678; 95%CI, 0.609 to 0.754; p<0.001) and showed a tendency for less surgery (OR, 0.894; 95%CI, 0.694 to 1.151; p = 0.384).

## Discussion

Consensus has been reached that TNBC is commonly associated with early age at diagnosis [4–7]. Accordingly evidence is far from sufficient regarding the survival pattern of elderly TNBC women (≥70 years) in comparison with the younger cases. In the current population study based upon SEER research data, 9908 TNBC patients were included and nearly one fifth of them were at advanced age. It was discovered that old-aged TNBC patients were more likely to develop tumors with lower histological grade, less lymph node metastasis and earlier TNM stage, yet counterintuitively exhibited increased early mortality. Age at diagnosis, together with loco-regional treatment status was demonstrated to be independent outcome-concerning variables in TNBC. Meanwhile it was uncovered that when contrasted with the young group local treatment encompassing surgery and radiotherapy was relatively inadequate in elderly cases, possibly contributing to a survival disadvantage in elderly TNBC, which was compromised after adjusting for the influence of therapy.

Older-aged individuals make up nearly 20% of TNBC cases [16]. Till now very little is known about this unique demographic group. Emerging evidence has created the impression that advanced age was associated with more favorable outcome in TNBC though the underlying mechanisms are mostly undetermined. It has been suggested that different distributions of certain biological variations might partly explain the age-related discrepancy in TNBC survival [18]. For example, *BRCA* mutations were more prevalent in young breast cancer patients and predicted poor prognosis [18, 19]. Yet most of the findings were derived from retrospective studies with small sample size, which needed to be verified in expanded sampled cohort. Contrary to previous belief, our population-based investigation concluded that elderly TNBC patients actually experienced elevated early mortality in comparison with the younger individuals. We speculate that such disagreement with existing research data was mainly due to the inconsistency in the follow-up duration as well as sample size. Considering that TNBC relapse events largely take place within the first 2–3 years of diagnosis, we focused on the short-term survival pattern instead of long-term prognosis. To our best knowledge, this is an initial attempt to capture a snapshot of TNBC immediate mortality status.

In the present study, older-aged women with TNBC displayed reduced OS compared with the younger individuals, which is consistent with previous finding that the risk of mortality increases with age. Naturally enough elderly patients tend to die of comorbidities such as cardiovascular diseases in addition to malignancy. There are two major aspects concerning the compromised CSS observed in older-aged TNBC. To start with, advanced age itself remained as an unfavorable prognostic element after ruling out potential interaction of confounders, even though old-aged tumors tended to be less biologically aggressive. Generally speaking, senility is usually connected with ill health condition and comorbidities that require additional care and monitoring, which will definitely complicate the clinical management of such cases and result in an increased risk of therapy-relevant adverse events. Moreover, it was found that compared with the younger women elderly individuals were less likely to receive loco-regional therapy, which was exemplified by a reduced proportion of patients undergoing surgery and radiotherapy. Surgery and radiotherapy were both indispensable treatment modalities especially in TNBC, a subtype naturally lacking molecular targets of endocrine therapy and chemotherapeutics. A remarkable finding derived from our study was that among patients with relatively advanced diseases (stage II or III) for whom surgery and postoperative radiotherapy should be indicated, use of loco-regional intervention especially radiotherapy was inadequate in elderly women, which coincided with the reduced CSS observed in this subgroup. Furthermore, surgery and radiation were demonstrated to be independent prognostic factors in both total TNBC population and the subset of elderly TNBC patients. Nevertheless we failed to eliminate the influence of confounding factors such as personal health status and receipt of systemic chemotherapy which are not recorded in SEER registries. Given the cohort nature of our study, we could only infer a possible association rather than establish a causal relationship between loco-regional undertreatment and poor immediate prognosis of elderly TNBC from the derived results. The driving elements that contribute to elevated early cancer-specific mortality in elderly patients with TNBC warrant further study.

Already known is that undertreatment in elderly breast cancer patients produced an eminent negative effect on survival [20–22]. Apart from surgical excision and radiotherapy, systematic chemotherapy in adjuvant setting has been proved to bring about noticeable benefit in OS and DFS of elderly breast cancer women, particularly in hormone receptor (HR)-negative cohorts. In a large retrospective study of approximately 6500 lymph node-positive breast cancer patients by the Cancer and Leukemia Group B (CALGB), it was revealed that elderly patients obtained similar decrease in risk of mortality and relapse to that of the younger women from adjuvant chemotherapy [23]. Another population-based study utilizing SEER-Medicare-linked database concluded that chemotherapy significantly reduced mortality in ER-negative lymph node-positive older women with breast cancer (≥70 years) [24]. The International Society of Geriatric Oncology (SIOG) recommended that in management of elderly patients with breast cancer the decision to perform surgery, adjuvant radiation and chemotherapy should not be based upon age alone. Other factors that should be taken into consideration include life expectancy, treatment tolerance, estimated benefit and personal preference [21, 22]. In spite of the established advantages of comprehensive treatment in older women with breast cancer, underuse of clinical intervention prevails among this rather heterogeneous population. In a SEER-based study of 6933 old-aged HR-negative breast cancer patients (≥80 years), Weiss et al found that older patients tended to receive less surgery and radiation [25]. A prospective Swiss study which was aimed to describe the treatment patterns of elderly patients with breast cancer (n = 4820) summarized that there existed substantial underuse of breast-conserving surgery (BCS), post-BCS radiotherapy, sentinel lymph node dissection (SLND) and adjuvant endocrine treatment in elderly patients (≥80 years) [26].

Potential reasons that might preclude the application of loco-regional treatment in older-aged individuals with TNBC are speculated. First, the study revealed that elderly patients were slightly more likely to have node-negative disease and T1 tumors, which may account for an appropriately lower rate of adjuvant radiotherapy. Furthermore, the decisions to forgo surgery or radiotherapy in older patients could be driven by valid concerns over post-therapy complications and even mortality. In a study evaluating surgery in older breast cancer patients (n = 449), Rocco et al concluded that advanced age (≥85 years) was associated with evidently higher risk of postoperative complications (OR, 5.75; 95%CI, 2.38 to 14.04; p< 0.001), which then had an undesirable bearing on OS (HR, 2.06; 95%CI, 1.52 to 2.70; p< 0.001) [27]. One prospective study involving 800 old-aged UK breast cancer patients determined that patients older than 75 years were less likely to undergo surgery (OR, 0.40; 95% CI, 0.22 to 0.73) mainly owing to poor physical condition [28]. Last but not least, personal preference is another essential factor that should be taken into consideration. As proved by our study, elderly patients were more inclined to refuse postoperative radiotherapy than younger women in spite of recommendation by their treating physicians.

The present study has several limitations. Above all, since crucial information concerning competing comorbidities and receipt of adjuvant chemotherapy is unavailable in SEER database, the observed association between loco-regional undertreatment and inferior outcome in elderly patients with TNBC could be confounded by external factors that are not recorded and thus cannot be established by the current cohort study. In order to solve this problem we set CSS as the primary outcome of our study considering that comorbidities such as cardiovascular diseases and hypertension exert a much more profound influence on OS than CSS. Admitting that the results of OS analysis (Table 2) were less reliable due to exclusion of comorbidity factors, we mainly focused on the impact of age on outcome of malignant disease. Besides, the valid reasons that account for the decreased proportion of surgery and radiotherapy in the older-aged group couldn’t be addressed because SEER database fails to provide relevant records. In addition, the subgroup analysis results (Fig. D in S2 File) should be interpreted with caution. In this unplanned subgroup analysis, lack of statistical significance may be due to insufficient power of the study which was evident from the relatively small number of events (71+21 = 92).

Admitting that geriatric patients are more vulnerable to the adverse impact of post-treatment complications due to comorbidities and functional deficiency of vital organs, denying standard anti-cancer treatment to this cohort merely based on age is not fully justified. However this dilemma still awaits more evidence from prospective clinical trials to be resolved since existing findings concerning the treatment of geriatric TNBC patients were all too limited and conflicting. Ongoing are clinical trials and studies that are designed to establish old-aged TNBC-specific treatment approaches. For instance, great efforts are made to identify subtypes of TNBC that require modified radical mastectomy with or without adjuvant radiation or BCS followed by radiation. Besides, the appropriate choice of chemotherapeutics and molecularly targeted agents in adjuvant settings among elderly TNBC women is being investigated.

## Conclusions

In our population-based analysis of 9908 women with TNBC, we found that elderly patients with TNBC had increased early mortality compared to the younger cases although they tended to develop tumors of lower grade and TNM stage. Advanced age at diagnosis (≥70 years) was significantly predictive of worse OS and CSS. These older patients with TNBC were less likely to receive loco-regional treatment encompassing surgery and radiation, which might be associated with the compromised cancer-specific survival observed in elderly TNBC.

## Supporting Information

S1 File Cancer-specific survival of elderly patients with TNBC stratified by surgery status (Figure A, 1 for surgery, 2 for no surgery) or radiation status (Figure B, 1 for radiation, 2 for no radiation). n, number of patients; O, observed events.
(TIF)

S2 File Cancer-specific survival of young (group 1) and elderly (group 2) patients with TNBC. Patients underwent neither surgery nor radiotherapy (Figure A, n = 460). Patients underwent surgery irrespective of radiation history (Figure B, n = 9324). Patients underwent surgery without radiotherapy (Figure C, n = 4354). Patients underwent both surgery and radiotherapy (Figure D, n = 4552). n, number of patients; O, observed events.
(TIF)

S1 Table Factors related to cancer-specific survival in elderly patients with triple-negative breast cancer by multivariate analysis. Cox proportional hazards regression model was used. Two-sided p-values were reported with p<0.05 considered statistically significant. Abbreviations: HR, hazard ratio.
(DOCX)
