# Supplementary figures and images for: Age-Related Disparity in Immediate Prognosis of Patients with Triple-Negative Breast Cancer: A Population-Based Study from SEER Cancer Registries
Source: PLoS One. 2015 May 28;10(5):e0128345. doi: 10.1371/journal.pone.0128345 (PMC4447406; doi:10.1371/journal.pone.0128345)

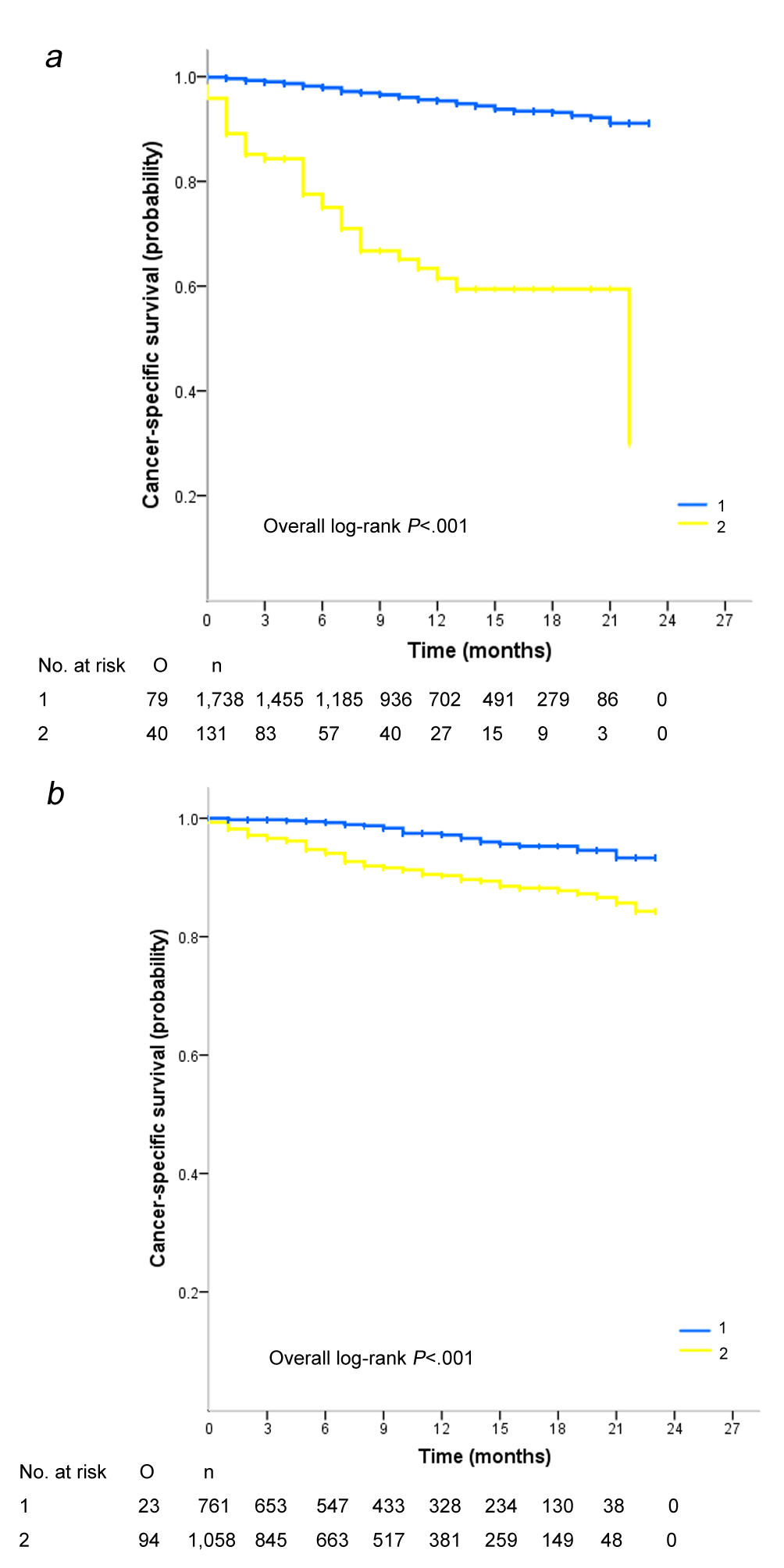

Supplement: S1 File — n, number of patients; O, observed events. (TIF) [file pone.0128345.s001.tif]

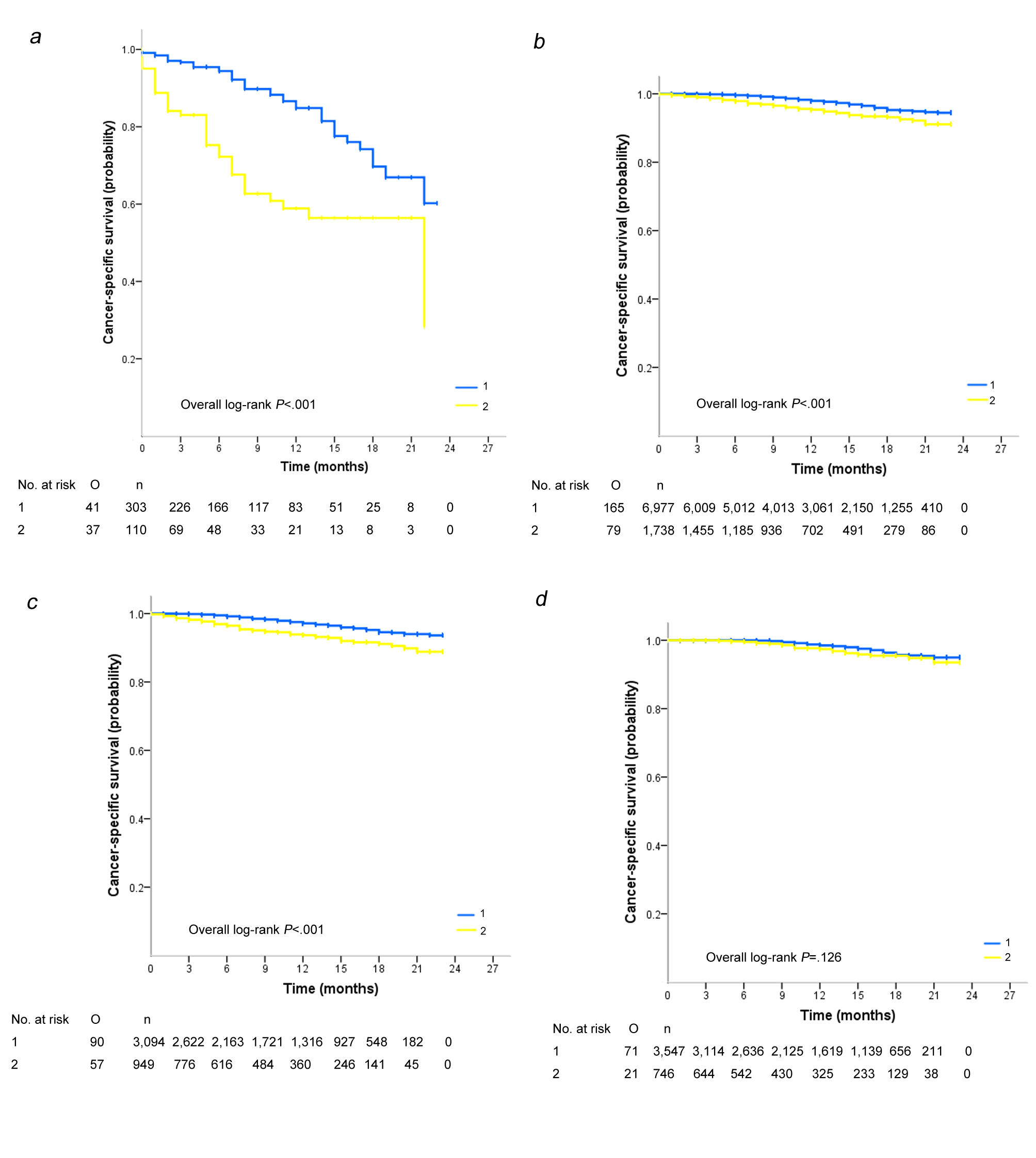

Supplement: S2 File — Patients underwent neither surgery nor radiotherapy (Figure A, n = 460). Patients underwent surgery irrespective of radiation history (Figure B, n = 9324). Patients underwent surgery without radiotherapy (Figure C, n = 4354). Patients underwent both surgery and radiotherapy (Figure D, n = 4552). n, number of patients; O, observed events. (TIF) [file pone.0128345.s002.tif]
