# Supplementary material for: Age-Related Disparity in Immediate Prognosis of Patients with Triple-Negative Breast Cancer: A Population-Based Study from SEER Cancer Registries
Source: PLoS One. 2015 May 28;10(5):e0128345. doi: 10.1371/journal.pone.0128345 (PMC4447406; doi:10.1371/journal.pone.0128345)
Supplement: S1 Table — Cox proportional hazards regression model was used. Two-sided p-values were reported with p<0.05 considered statistically significant. Abbreviations: HR, hazard ratio. (DOCX) [file pone.0128345.s003.docx]

**S1 Table. Factors related to cancer-specific survival in elderly patients with triple-negative breast cancer by multivariate analysis.**

|  | **HR** | **p value** |
| --- | --- | --- |
|  |  |  |
| **Surgery** |  | **<0.001** |
| **No** | — |  |
| **Yes** | 0.250 |  |
| **Radiation** |  | **<0.001** |
| **No** | — |  |
| **Yes** | 0.402 |  |
| **Grade** |  | 0.402 |
| **I-II** | — |  |
| **III-IV** | 1.263 |  |
| **TNM stages** |  | **0.008** |
| **I** | — |  |
| **II-III** | 2.807 |  |
| **Tumor size** |  | **<0.001** |
| **T0-2** | — |  |
| **T3-4** | 3.181 |  |
| **Node metastasis** |  | **0.004** |
| **N0** | — |  |
| **≥N1** | 1.918 |  |

Cox proportional hazards regression model was used. Two-sided p-values were reported with p<0.05 considered statistically significant. Abbreviations: HR, hazard ratio.
